# Giant Pancreatic Mucinous Cystadenoma Treated by Enucleation: A Case Report and Review of the Literature

**DOI:** 10.1002/ccr3.73732

**Published:** 2026-10-20

**Authors:** Ghofran Mahgoub Hussien, Nassir Alhaboob Arabi, Abdulmagid Mohammed Musaad, Elsaggad Eltayeb Mohamed, Sawsan Abulgasem Mohamed, Tarig Gasim Mohamed Alarabi

**Affiliations:** ^1^ Department of Gastrointestinal and Hepatopancreatobiliary Surgery Ibn Sina Specialized Hospital Khartoum Sudan; ^2^ Faculty of Medicine, Department of Gastrointestinal and Hepatopancreatobiliary Surgery Omdurman Islamic University Khartoum Sudan; ^3^ Faculty of Medicine Omdurman Islamic University Khartoum Sudan; ^4^ Department of Gastrointestinal Surgery Ibn Sina Specialized Hospital Khartoum Sudan; ^5^ Faculty of Medicine Al Neelain University Khartoum Sudan; ^6^ Department of Anatomy College of Medicine, King Khalid University Abha Saudi Arabia

**Keywords:** cystadenoma, cystic tumor, enucleation, pancreas, resection

## Abstract

A giant pancreatic mucinous cystic neoplasm in an elderly man was successfully enucleated with an uneventful recovery. This case emphasizes careful differentiation of pancreatic cystic lesions and individualized surgical planning; in selected patients without features suspicious for malignancy, enucleation may preserve pancreatic tissue while achieving complete removal.

## 1 Introduction

Pancreatic cystic neoplasms are uncommon and are often discovered incidentally, with a reported prevalence of approximately 0.7%–2.5% on computed tomography (CT) and magnetic resonance imaging (MRI) [1, 2]. Mucinous cystic neoplasms (MCNs) occur predominantly in middle‐aged women and usually arise in the body or tail of the pancreas [3]. Serous and mucinous cystadenomas are among the more common benign pancreatic cystic neoplasms [4]. MCNs generally do not communicate with the main pancreatic duct and may be unilocular or multilocular; giant lesions are uncommon.

Large pancreatic cystic lesions can be difficult to distinguish from malignant cysts and may prompt formal pancreatic resection. However, when features suspicious for malignancy, such as mural nodules or calcification, are absent, a parenchyma‐preserving procedure may be considered in selected patients.

The present case is unusual because a giant pancreatic tail MCN occurred in a 68‐year‐old man, whereas MCNs are reported predominantly in middle‐aged women. The lesion was also successfully removed by enucleation despite its exceptional size, making the case relevant to individualized surgical decision‐making for large pancreatic cystic neoplasms.

## 2 Case Presentation/Examination

A 68‐year‐old man presented with a 3‐year history of dull, aching upper abdominal pain that neither shifted nor radiated and had no clear relieving or aggravating factors. The pain was associated with heartburn and dyspepsia. His appetite was preserved, with no weight loss. There were no other gastrointestinal symptoms, no previous history of jaundice, and no features suggestive of acute pancreatitis. He was neither diabetic nor hypertensive. He had undergone left inguinal hernia repair 10 years earlier. He reported no smoking or alcohol consumption.

General and abdominal examinations were unremarkable. No palpable abdominal mass was detected.

## 3 Differential Diagnosis, Investigations, and Treatment

The differential diagnosis included pancreatic mucinous cystic neoplasm, pancreatic pseudocyst, serous cystadenoma, and intraductal papillary mucinous neoplasm. Abdominal ultrasonography showed a huge cystic mass of indeterminate origin. Contrast‐enhanced CT of the abdomen with oral and intravenous contrast demonstrated a huge unilocular cyst arising from the pancreatic tail and extending inferiorly toward the left kidney and pelvis (Figure 1). A further CT image demonstrated the relationship of the cyst to the pancreatic tail (Figure 2). The liver was normal, with no ascites or lymphadenopathy. Serum amylase, carcinoembryonic antigen (CEA), and carbohydrate antigen 19‐9 (CA 19‐9) were within normal limits.

**FIGURE 1:**
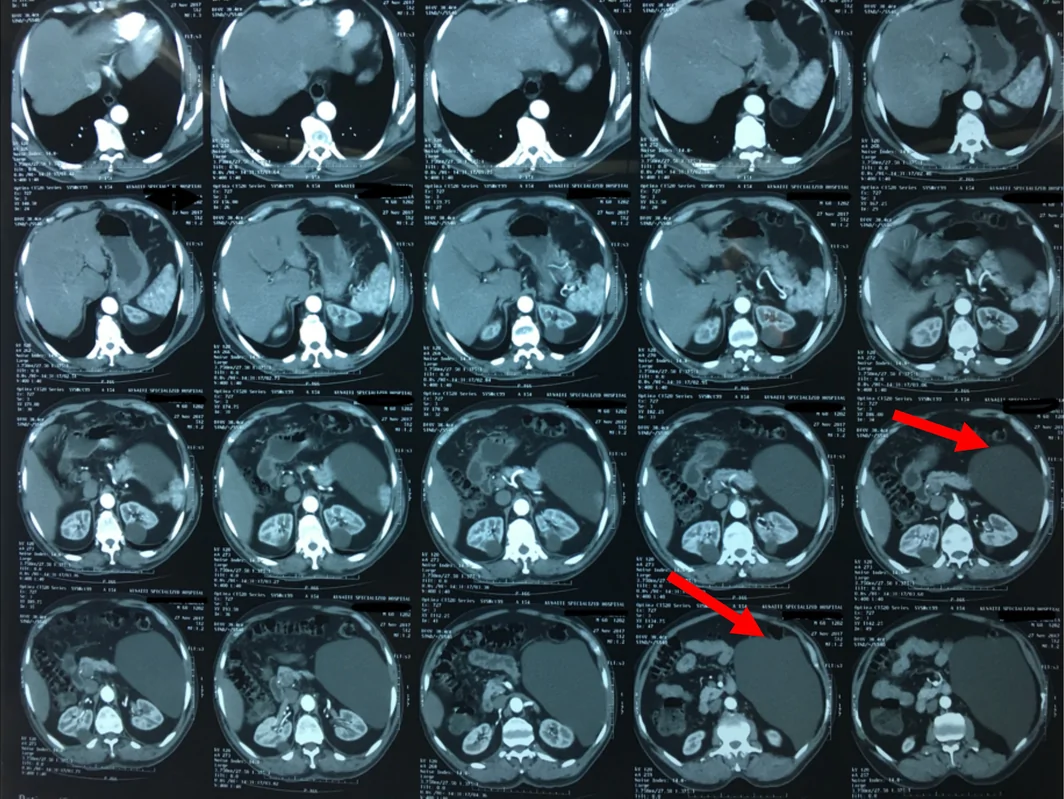
Contrast‐enhanced computed tomography of the abdomen with oral and intravenous contrast demonstrating a large cystic lesion arising from the distal pancreas. The red arrows indicate the pancreatic cyst.

**FIGURE 2:**
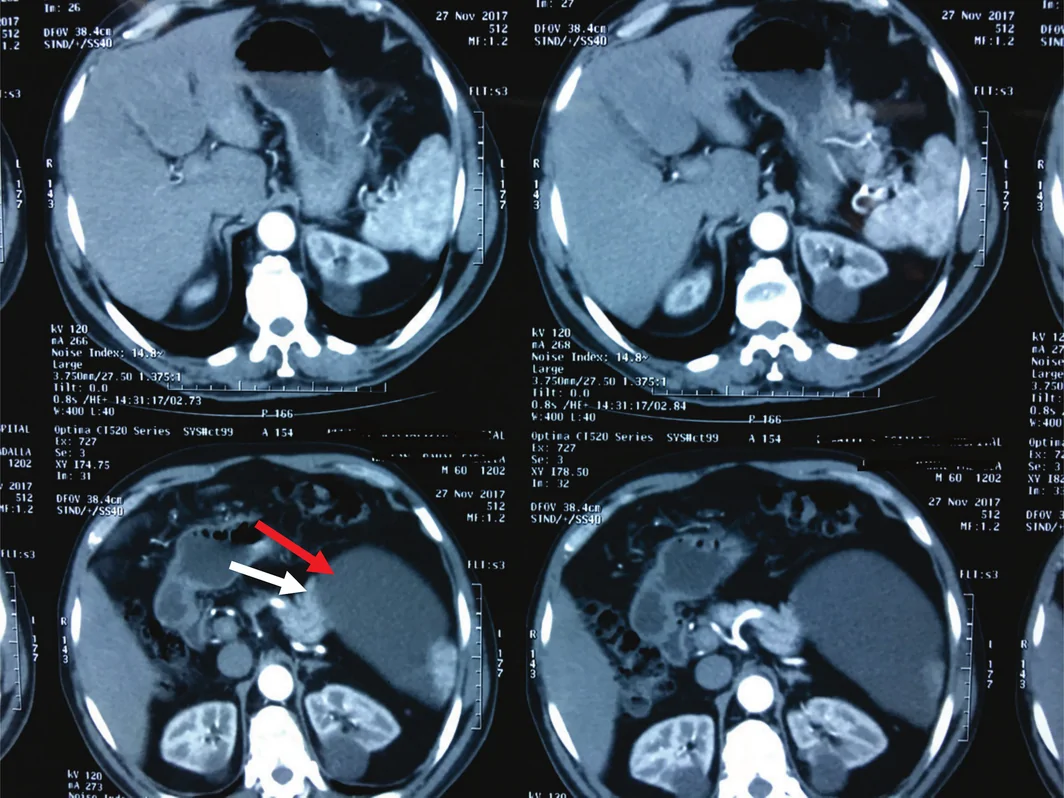
Contrast‐enhanced computed tomography of the abdomen with oral and intravenous contrast demonstrating the anatomical relationship between the lesion and the pancreatic tail. The red arrow indicates the pancreatic cyst, and the white arrow indicates the tail of the pancreas.

Exploratory laparotomy revealed a large retrogastric cystic mass arising from the pancreatic tail and extending laterally toward the left kidney and inferiorly into the pelvis. Careful sharp and blunt dissection was performed to release the medial and lateral adhesions. Enucleation was completed successfully, and the mass was removed intact. The cyst measured approximately 24 × 17 cm (Figure 3). Blood loss was minimal, and a surgical drain was placed. Cyst‐fluid analysis showed normal amylase and CA 19‐9 levels, no bacterial growth, and negative cytology. Histopathological examination confirmed mucinous cystadenoma.

**FIGURE 3:**
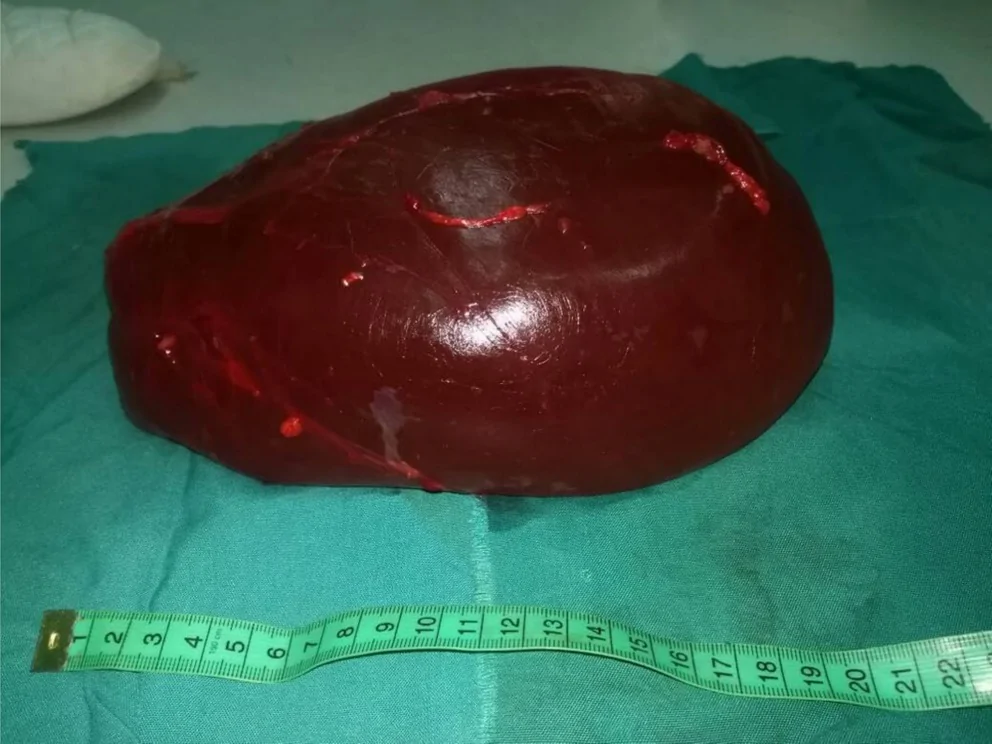
Photograph of the intact giant pancreatic cyst after successful enucleation. The specimen measured approximately 24 × 17 cm.

## 4 Conclusion and Results (Outcome and Follow‐Up)

The postoperative course was uneventful. The surgical drain was removed on postoperative Day 4, and the patient was discharged home in good condition. At follow‐up, the patient remained asymptomatic with no evidence of recurrence. This case demonstrates that enucleation can be performed successfully in a selected patient with a giant MCN when there is no clinical, radiologic, or intraoperative suspicion of malignancy.

## 5 Discussion

Pancreatic cystic tumors are uncommon and have been reported to constitute approximately 10%–15% of pancreatic masses and 1%–10% of pancreatic malignancies [5, 6]. They are often slow‐growing and may remain asymptomatic until they become large. In the absence of trauma or acute or chronic pancreatitis, a cystic pancreatic neoplasm should be considered rather than assuming a pseudocyst, although differentiation can be difficult [7]. Pancreatic cystic tumors include mucinous cystic neoplasms, serous cystic neoplasms, intraductal papillary mucinous neoplasms, solid pseudopapillary neoplasms, and other rare lesions [8]. MCNs usually measure 1.5–10 cm, although lesions up to 35 cm have been reported [9, 10]. The present 24 × 17 cm lesion therefore represents an uncommon giant MCN, and its occurrence in a 68‐year‐old man contrasts with the marked female predominance described in the literature [3, 10].

With widespread use of high‐quality imaging, pancreatic cystic neoplasms are increasingly detected. MRI and CT have comparable diagnostic performance for differentiating benign and malignant pancreatic cystic lesions, while endoscopic ultrasound (EUS) is useful for guided sampling [11, 12, 13]. CEA can assist in identifying mucinous cysts, but a normal value does not exclude an MCN [14]. In this patient, CT demonstrated a large unilocular pancreatic tail cyst without reported ascites or lymphadenopathy, and serum amylase, CEA, and CA 19–9 were normal. These findings supported a benign cystic process but did not replace definitive histopathological diagnosis.

The reported risk of invasive carcinoma in MCNs is approximately 7%–12%, and features associated with greater concern include size > 4 cm, mural nodules, and calcification [15]. Surgical management depends on lesion type, symptoms, malignant potential, growth, location, and technical considerations [16]. Resection has been advocated for symptomatic large lesions, lesions with malignant potential, and lesions showing rapid growth [1, 17, 18]. Although the size of the lesion in this patient was concerning, no additional clinical or radiologic features suspicious for malignancy were reported, and histopathology ultimately confirmed mucinous cystadenoma.

Pancreatic enucleation may preserve functioning pancreatic tissue and avoid some morbidity associated with major pancreatic resection. Previous reports have described favorable outcomes with enucleation in selected benign or low‐grade pancreatic lesions [7, 19, 20]. In the present case, complete enucleation of a 24 × 17 cm lesion was achieved, followed by an uneventful postoperative course. This case adds to the literature by showing that, in a carefully selected patient, giant size alone does not necessarily preclude a parenchyma‐preserving operation when malignancy is not suspected and complete removal can be achieved safely.

## Author Contributions

**Ghofran Mahgoub Hussien:** conceptualization, data curation, investigation, methodology, project administration, resources. **Nassir Alhaboob Arabi:** conceptualization, data curation, investigation, methodology, project administration, resources. **Abdulmagid Mohammed Musaad:** conceptualization, data curation, formal analysis, software, supervision, writing – review and editing. **Elsaggad Eltayeb Mohamed:** conceptualization, data curation, formal analysis, investigation, software, supervision, writing – review and editing. **Sawsan Abulgasem Mohamed:** formal analysis, validation, visualization, writing – original draft. **Tarig Gasim Mohamed Alarabi:** writing – original draft, writing – review and editing.

## Funding

The authors have nothing to report.

## Consent

Written informed consent was obtained from the patient for publication of this case report and accompanying images in accordance with journal guidelines.

## Conflicts of Interest

The authors declare no conflicts of interest.

## Data Availability

Data supporting the findings of this case report are included in the article. Additional anonymized data that do not compromise the patient's identity are available from the corresponding author upon reasonable request and subject to patient consent and applicable ethical requirements.
